# DNA damage response associated miRNA dysregulation in Ataxia-telangiectasia

**DOI:** 10.1007/s11033-026-12860-1

**Published:** 2026-09-30

**Authors:** Muhammad Junaid Iqbal, Cecilia Bucci, Anastasia Ricci, Gianluca Morganti, Faez Iqbal Khan, Muhammad Jawad Khan, Michele Menotta

**Affiliations:** 1 Department of Biomolecular Sciences, University of Urbino “Carlo Bo”, Via Saffi 2, 61029 Urbino (PU), Italy; 2 Centre of Excellence for Oncology and Immunology, Academy of Life and Natural Sciences, Xi’an Jiaotong-Liverpool University, No. 111 Ren’ai Road, Suzhou, 215123 China; 3 Department of Biosciences, COMSATS University Islamabad, Park Road, Tarlai Kalan, Islamabad, 45550 Pakistan

**Keywords:** ATM, miR-34a-5p, miR-26a-5p, Ataxia Telangiectasia, DNA Damage, MicroRNA

## Abstract

**Background:**

Ataxia-telangiectasia (A-T) is a rare autosomal recessive genome-instability disorder due to pathogenic variants in ATM, a central kinase of the DNA double-strand break signaling, checkpoint activation and stress-response pathways. Although microRNAs (miRNAs) regulate DNA damage response (DDR), apoptosis, senescence and cell-cycle control, the stress-responsive behavior of selected ATM/DDR-associated miRNAs remains incompletely characterized in A-T fibroblast models.

**Methods and Results:**

Four predefined ATM/DDR-associated candidate miRNAs (hsa-miR-34a-5p, hsa-miR-26a-5p, hsa-miR-106b-5p and hsa-miR-20a-5p) were quantified in one ATM-proficient fibroblast control and three A-T fibroblast cell lines both under basal and bleomycin exposed conditions. The relative expression of selected miRNAs was measured by RT-qPCR. miR-34a-5p and miR-26a-5p levels were lower in each of the three bleomycin-treated A-T lines relative to the single bleomycin-treated WT control line. miR-34a-5p and miR-26a-5p levels were lower in bleomycin-treated three A-T lines relative to the single control WT line. Within-cell-line analysis showed significant miR-34a-5p induction after bleomycin in WT, AT648 and AT9607, whereas significant miR-26a-5p induction was observed only in AT648. AT648 showed the broadest within-cell-line response, with significant induction of all four tested miRNAs after bleomycin. Target-based enrichment and network analyses of the predefined candidate panel identified annotations related to cell-cycle regulation, G1/S transition, RB/E2F signaling, p53 signaling, apoptosis, senescence, PI3K-Akt signaling, and DNA damage-associated processes. Network analysis revealed a number of shared candidate targets such as *CCND1*, *CCND2*, *E2F1*, *E2F3*, *RB1*, *WEE1*, *PTEN*, *SMAD4*, *MYC* and *VEGFA*.

**Conclusions:**

In this fibroblast panel, miR-34a-5p and miR-26a-5p showed the most consistent baseline alterations, whereas bleomycin responsiveness was cell-line specific and heterogeneous.

**Supplementary Information:**

The online version contains supplementary material available at https://doi.org/10.1007/s11033-026-12860-1.

## Introduction

Ataxia-telangiectasia (A-T) is a rare autosomal recessive genome-instability disorder that is characterized by progressive neurodegeneration of the cerebellum, the presence of oculocutaneous telangiectasia, immunodeficiency, radiosensitivity, recurrent infections and increased cancer susceptibility [1, 2]. A-T is caused by biallelic pathogenic variants in *ATM*, which encodes a large serine/threonine kinase of the phosphatidylinositol 3-kinase-related kinase family [3, 4]. ATM is a key regulator in the DNA double-strand break (DSB) response. The ATM is recruited and activated upon DSBs via damage sensing complexes, and phosphorylates several downstream targets including CHK2, p53, H2AX, NBS1 and BRCA1, which are involved in checkpoint control, DNA repair signaling, apoptosis and senescence. In addition to canonical DSB signaling, ATM is also involved in responding to oxidative stress, mitochondrial homeostasis and cellular stress adaptation [4, 5].

MicroRNAs (miRNAs) are small non-coding RNAs that control the expression of their target genes post-transcriptionally by destabilising mRNA or preventing the translation of mRNA into proteins [6]. In the DNA damage response (DDR), miRNAs modulate repair, checkpoint control, apoptosis, senescence and cell-cycle progression [7, 8]. ATM might affect miRNA biology on multiple levels, as damage-responsive miRNAs can be transcriptionally regulated through p53, miRNAs can be activated by ATM, and miRNA biogenesis can be promoted by ATM. However, Zhang et al. demonstrated that ATM activation could lead to global miRNA biogenesis in an ATM-dependent manner by phosphorylation of KSRP which connects ATM signaling directly to miRNA processing [9]. Regulation between ATM and miRNAs can also occur in the opposite direction. Hu et al. demonstrated that miR-421 can suppress ATM expression by targeting the ATM 3′UTR, resulting experimentally in checkpoint defects and increased radiosensitivity [10]. In this context, we chose a panel of only candidate miRNAs that were selected to assess complementary arms of the ATM-associated DDR and checkpoint network. miR-34a-5p was selected because miR-34a is a canonical p53-responsive miRNA, which is involved in apoptosis, cell-cycle arrest and senescence [11–14], and also mature miR-34 activity can be modulated through ATM-dependent 5′-end phosphorylation after DNA damage [15]. miR-26a-5p was selected because it has been linked to genotoxic stress and checkpoint regulation, including reported regulation of WEE1 and CHK1, as well as DNA repair and PTEN-AKT-associated phenotypes in cancer models [16–18]. miR-106b-5p and miR-20a-5p were included to represent the G1/S and RB/E2F checkpoint-modulatory arm: miR-106b family members regulate *CDKN1A*/p21 and promote cell-cycle progression [19], while miR-20a participates in c-Myc/E2F1-associated G1 checkpoint control [20]. Related miRNAs from the miR-17, miR-20a and miR-106b families can also act together on overlapping cell-cycle targets [21].

However, there is limited evidence of dysregulation of non-coding RNAs in A-T. Cirillo et al. recently profiled miRNAs in A-T PBMCs and fibroblasts and validated the changes of miR-342-3p, miR-30a-5p and miR-195-5p in the disease [22]. However, there is still limited information on the specific role of ATM, p53 and G1/S-related candidate miRNAs in response to controlled genotoxic stress. As such, this panel was not designed as an unbiased biomarker screen, but rather as a mechanistically based assay of miRNAs at the nexus of ATM/p53 signaling and DNA damage response and G1/S checkpoint control. In this study, we measured the expression of miR-34a-5p, miR-26a-5p, miR-106b-5p and miR-20a-5p in one ATM-proficient fibroblast control and three A-T fibroblast cell lines in basal conditions and following bleomycin exposure. Bleomycin is a radiomimetic glycopeptide that generates oxidative DNA lesions and DNA strand breaks through an iron- and oxygen-dependent mechanism. Because ATM is a central transducer of DNA double-strand-break signaling, bleomycin was selected as a controlled genotoxic stimulus to compare basal and stress-responsive behavior of the predefined candidate miRNA panel in the studied fibroblast lines [23, 24].

## Materials and methods

### Cell lines and experimental design

One *ATM*-proficient human fibroblast control line, AG09429, and three patient-derived A-T fibroblast cell lines, GM00648, GM05849 and GM09607, were used in this study. All lines were obtained from the Coriell Institute for Medical Research, NJ USA. The AG09429 was derived from gingival fibroblasts from an apparently healthy female donor and was used as the WT control. Cell lines GM00648, GM05849 and GM09607 were A-T fibroblast lines and are referred to as AT648, AT5849 and AT9607, respectively. However, AG09429 and GM00648 were originally untransformed and were immortalized in-house by hTERT transduction using the hTERT cell immortalization kit (Alstem Cell Advancements), as previously described [25]. Both GM05849 and GM09607 were obtained as transformed fibroblast lines, with GM05849 reported as SV40-transformed and GM09607 reported as pSV plasmid-transformed. Cells were maintained in Minimum Essential Medium Eagle supplemented with 2 mmol/L L-glutamine (Sigma-Aldrich), 100 U/mL penicillin (Sigma-Aldrich), 0.1 mg/mL streptomycin, 15% fetal bovine serum (Thermo Fisher Scientific) and 10 mM glucose. Cells were grown at 37 °C in a humidified atmosphere containing 5% CO_2_.

### Bleomycin treatment

DNA damage was induced using bleomycin sulphate (Alfa Aesar, J60727) [23, 24]. For each cell line, parallel cultures were either left untreated or treated with bleomycin at a final concentration of 8 µg/mL in complete medium for 3 h at 37 °C. After treatment, the bleomycin-containing medium was removed, cells were rinsed with phosphate-buffered saline and incubated for a further 24 h in fresh complete medium before RNA extraction. Untreated cultures were processed in parallel as time-matched controls.

### RNA extraction

Total RNA was isolated using the miRNeasy Mini Kit (Qiagen) at the end of the 24 h recovery period or at the matched untreated time point. RNA concentration and purity were assessed using a NanoDrop 2000 spectrophotometer. Samples with A260/A280 > 1.8 were included in downstream analyses.

### Reverse transcription and qPCR

Relative changes in miRNA abundance were quantified using TaqMan MicroRNA Assays (Applied Biosystems, Thermo Fisher Scientific, USA). The following assays were used: hsa-miR-34a-5p, assay ID 000426; hsa-miR-26a-5p, assay ID 000405; hsa-miR-106b-5p, assay ID 000442; and hsa-miR-20a-5p, assay ID 000580. The hsa-miR-16-5p, assay ID 000391 was used as internal control for data normalization. For reverse transcription, 10 ng of total RNA was used for each reaction in a final volume of 5 µL using miRNA-specific stem-loop primers on a GeneAmp PCR System 2700. Reverse transcription was performed using the TaqMan MicroRNA Reverse Transcription Kit (Applied Biosystems, Thermo Fisher Scientific, USA). The thermal profile consisted of 16 °C for 30 min, 42 °C for 30 min and 85 °C for 5 min, followed by a 4 °C hold. Quantitative PCR was performed in technical duplicate on a QuantStudio 5 Real-Time PCR System and mean Ct values were used for downstream analysis. The qPCR cycling conditions were 95 °C for 10 min, followed by 40 cycles of 95 °C for 15 s and 60 °C for 60 s. Differences in miRNA expression were calculated using the comparative Ct method [26]. Ct values were normalized to hsa-miR-16-5p as the endogenous control. For baseline WT versus A-T comparisons, untreated WT was used as the calibrator. For bleomycin-treated WT versus A-T comparisons, bleomycin-treated WT was used as the calibrator. For within-cell-line bleomycin-response analysis, the corresponding untreated condition of each cell line was used as the calibrator. Relative expression was calculated using the 2^-ΔΔCt^ method and presented as fold change.

### Target-based pathway enrichment and miRNA-target network analysis

Target-based functional enrichment analysis was performed using DIANA-miRPath v4.0 in miRNA-centric mode [27]. The input list included hsa-miR-34a-5p, hsa-miR-26a-5p, hsa-miR-106b-5p and hsa-miR-20a-5p. hsa-miR-16-5p was excluded because it was used as the endogenous normalizer. *Homo sapiens* was selected as the organism, and miRTarBase 2022 was used as the experimentally validated miRNA-target interaction resource [28]. Enrichment was performed for KEGG, Reactome, Gene Ontology Biological Process and MSigDB Hallmark gene sets. Terms with Benjamini-Hochberg FDR-adjusted *p* < 0.05 were considered significant. For visualization, significant terms relevant to DNA damage response, cell-cycle regulation, checkpoint control, apoptosis, senescence and survival signalling were prioritized, while full unfiltered enrichment outputs were retained in the supplementary tables. DIANA-TarBase v8.0 was additionally used as a sensitivity interaction resource to assess whether the major pathway-level findings were retained when an alternative experimentally supported miRNA-target database was used. Because the four miRNAs constituted a predefined candidate panel selected partly on the basis of their reported involvement in p53, cell-cycle, checkpoint, and DNA damage-associated biology, enrichment analysis was used to characterize biological processes represented among their documented targets rather than as an independent validation of the RT-qPCR findings.

Network-based miRNA-target analysis was performed using MIENTURNET [29]. The same four candidate miRNAs were submitted, and miRTarBase was selected as the interaction source. The resulting miRNA-target interaction table was exported and visualized in Cytoscape (version 3.10.4) [30]. For final visualization, target genes shared by at least two input miRNAs were retained. MIENTURNET functional enrichment outputs were used as a complementary pathway-level assessment of the shared target network.

### Statistical analysis

Statistical analysis was performed using GraphPad Prism (version 9.1). All statistical tests were performed on ΔCt. For comparisons between WT and A-T cell lines within each condition, one-way ANOVA followed by Dunnett’s multiple-comparison test was used, with WT as the control group. For untreated versus bleomycin-treated comparisons within each cell line, paired Student’s *t*-tests were performed using matched biological replicates. Data were presented as mean ± SD from three independent biological replicates. Statistical significance was defined as *p* < 0.05. Figure annotations were assigned as *p < 0.05, **p < 0.01, and ***p < 0.001.

## Results

### Basal A-T fibroblast lines show marked depletion of miR-34a-5p and miR-26a-5p

**Fig. 1:**
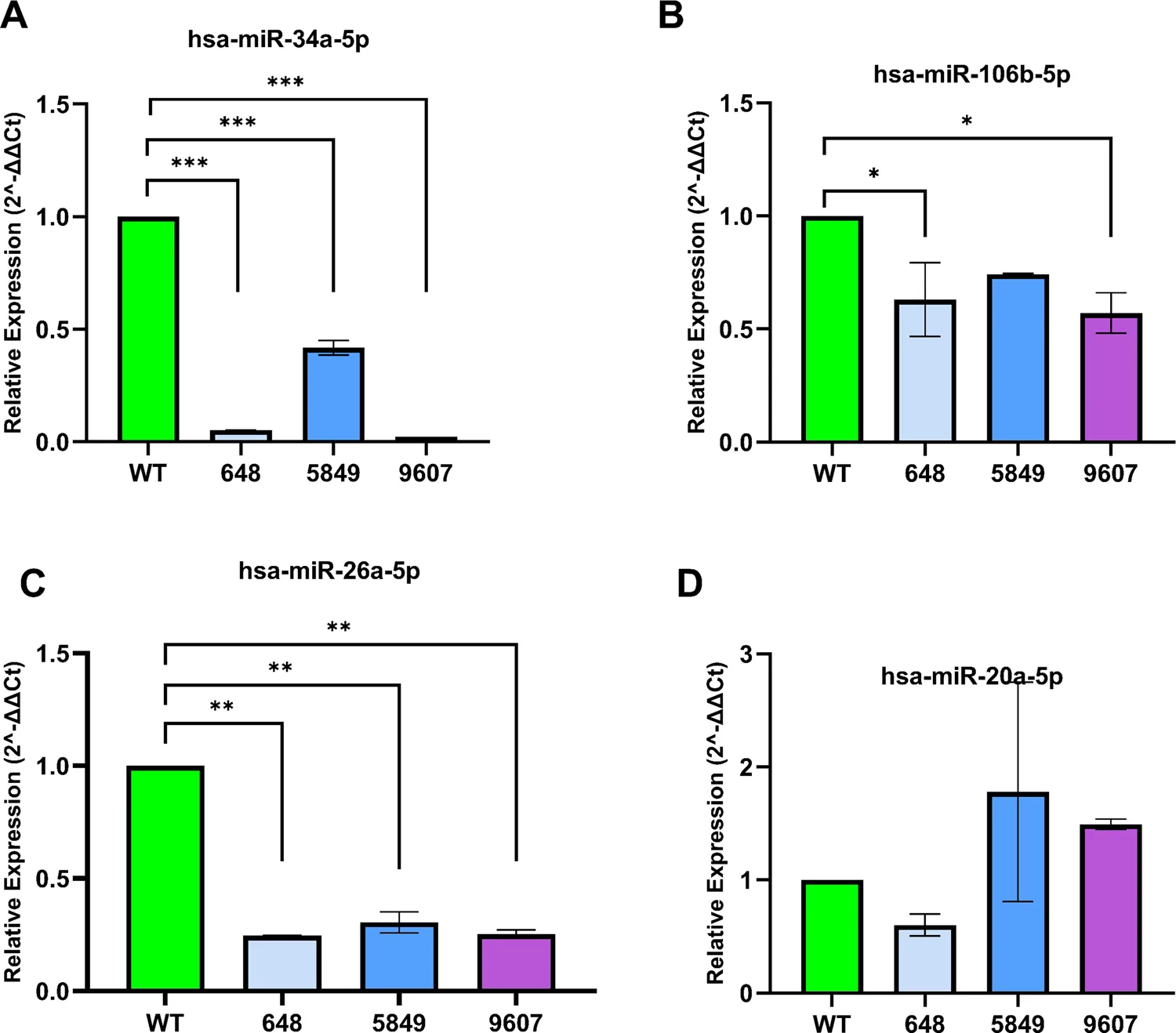
Basal expression of candidate DDR-associated miRNAs in WT and A-T fibroblast lines. Relative expression of **(A)** hsa-miR-34a-5p, **(B)** hsa-miR-106b-5p, **(C)** hsa-miR-26a-5p and **(D)** hsa-miR-20a-5p was assessed in untreated WT, AT648, AT5849 and AT9607 fibroblast lines. Expression levels were normalized to hsa-miR-16-5p and presented relative to untreated WT cells. Statistical analysis was performed on ΔCt values using one-way ANOVA followed by Dunnett’s multiple-comparison test against WT. Bars represent mean ± SD from three independent biological replicates. *p < 0.05, **p < 0.01, ***p < 0.001; ns, not significant

Under basal conditions, miR-34a-5p showed the most pronounced difference between WT and A-T fibroblast panel (Fig. 1A). Compared with untreated WT, all three A-T lines showed a significant reduction in miR-34a-5p expression after Dunnett’s multiple-comparison testing, AT648, *p* < 0.001; AT5849, *p* < 0.001; AT9607, *p* < 0.001. miR-26a-5p showed a similar pattern, with significantly lower expression in all three A-T lines compared with WT (Fig. 1C), AT648, *p* = 0.001; AT5849, *p* = 0.003; AT9607, *p* = 0.001. Thus, miR-34a-5p and miR-26a-5p represented the most consistent basal alterations across the A-T fibroblast panel. miR-106b-5p showed a weaker and more line-dependent basal profile (Fig. 1B). Expression was significantly reduced in AT648, *p* = 0.03, and AT9607, *p* = 0.01, but not in AT5849, *p* = 0.18. By contrast, miR-20a-5p did not differ significantly between WT and any A-T line under basal conditions (Fig. 1D), AT648, *p* = 0.21; AT5849, *p* = 0.32; AT9607, *p* = 0.37. Overall, basal A-T fibroblast lines showed a dominant reduction of miR-34a-5p and miR-26a-5p, while miR-106b-5p showed partial line-specific reduction and miR-20a-5p did not show a robust basal A-T-associated pattern. Full statistical outputs for baseline comparisons are provided in Supplementary Table S1A.

### miR-34a-5p and miR-26a-5p remain reduced in A-T fibroblast lines after bleomycin exposure

**Fig. 2:**
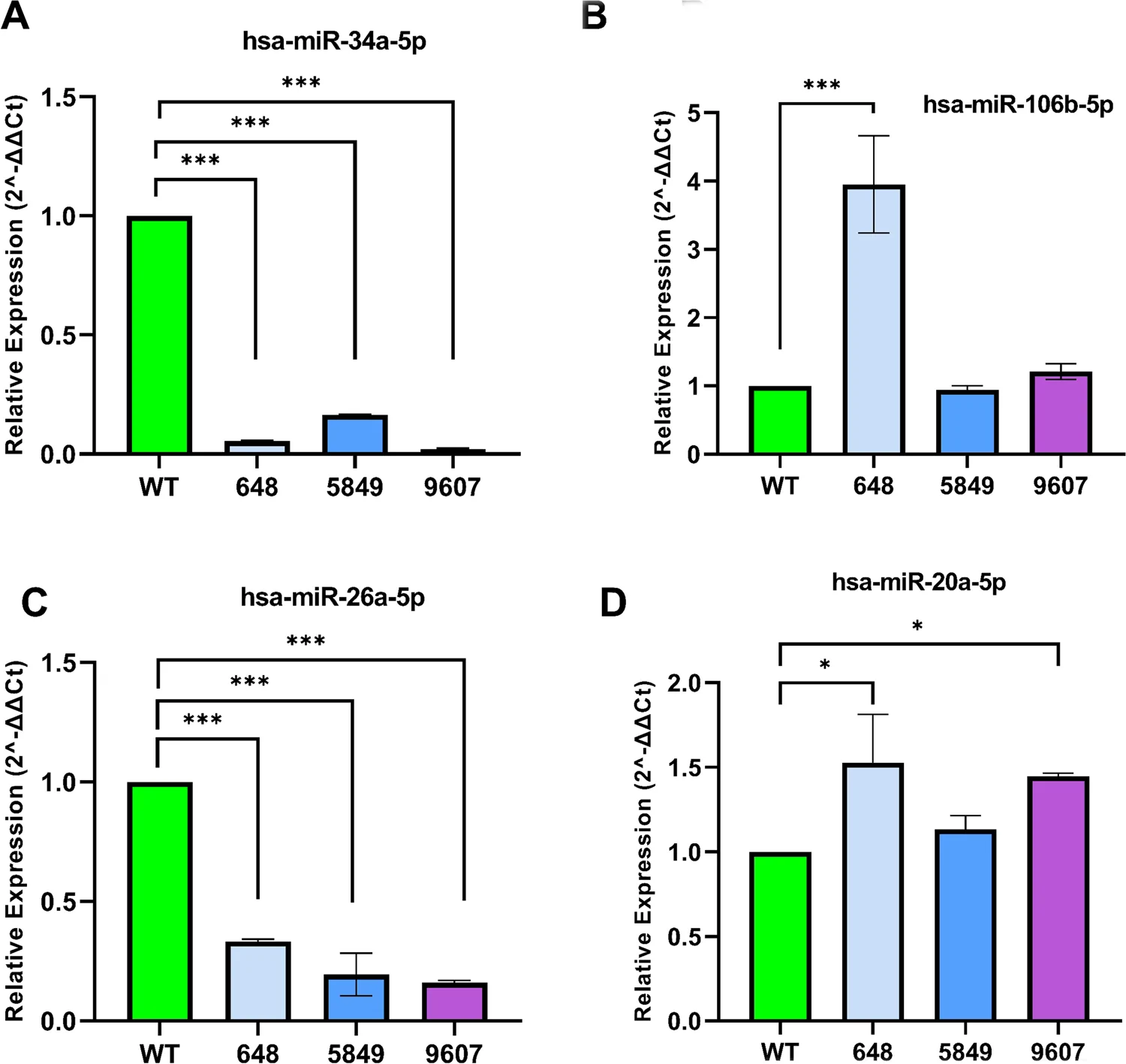
Candidate miRNA expression in bleomycin-treated WT and A-T fibroblast lines. Relative expression of **(A)** hsa-miR-34a-5p, **(B)** hsa-miR-106b-5p, **(C)** hsa-miR-26a-5p and **(D)** hsa-miR-20a-5p was assessed in bleomycin-treated WT, AT648, AT5849 and AT9607 fibroblast lines. Expression levels were normalized to hsa-miR-16-5p and presented relative to bleomycin-treated WT cells. Statistical analysis was performed on ΔCt values using one-way ANOVA followed by Dunnett’s multiple-comparison test against bleomycin-treated WT cells. Bars represent mean ± SD from three independent biological replicates. *p < 0.05, **p < 0.01, ***p < 0.001; ns, not significant

Next, we assessed whether the basal miRNA differences persisted after bleomycin exposure. In bleomycin-treated cells, miR-34a-5p remained significantly lower in all three A-T lines compared with single bleomycin-treated WT line (Fig. 2A), AT648, *p* < 0.001; AT5849, *p* < 0.001; AT9607, *p* < 0.001. The same pattern was observed for miR-26a-5p (Fig. 2C). All three A-T lines showed significantly reduced miR-26a-5p expression compared with bleomycin-treated WT, AT648, *p* < 0.001; AT5849, *p* < 0.001; AT9607, *p* < 0.001. These comparisons show that the between-line differences in miR-34a-5p and miR-26a-5p remained detectable after bleomycin exposure. However, persistence of a between-line difference does not itself indicate an acute treatment response, which was evaluated separately by the within-cell-line analyses. In contrast, miR-106b-5p showed a distinct post-bleomycin pattern (Fig. 2B). AT648 displayed significantly higher miR-106b-5p expression than bleomycin-treated WT, *p* < 0.001, whereas AT5849 and AT9607 did not differ significantly from WT, *p* = 0.91 and *p* = 0.27, respectively. miR-20a-5p also showed condition-dependent behaviour after bleomycin treatment (Fig. 2D). Expression was significantly higher in AT648, *p* = 0.03, and AT9607, *p* = 0.04, compared with bleomycin-treated WT, while AT5849 remained unchanged, *p* = 0.65. Together, the bleomycin-treated comparison shows that the three A-T lines retain lower miR-34a-5p and miR-26a-5p expression relative to the single control, while miR-106b-5p and miR-20a-5p show more condition-dependent and line-specific behaviour, with the strongest divergence observed in AT648. Full statistical outputs for bleomycin-treated comparisons are provided in Supplementary Table S1B.

### Bleomycin induces a cell-line-specific miRNA response rather than a uniform A-T profile

**Fig. 3:**
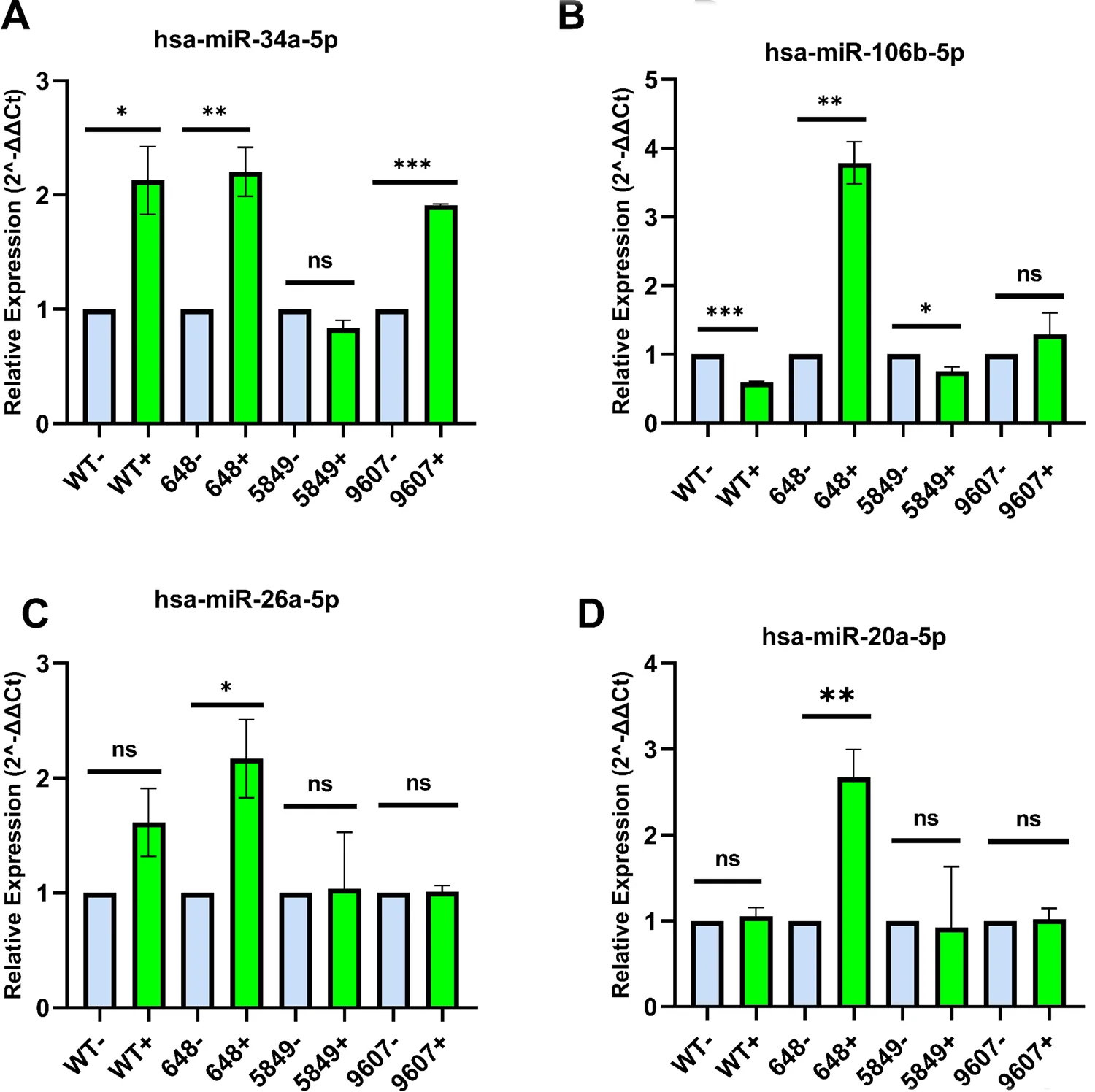
Within-cell-line miRNA response to bleomycin exposure. Within-cell-line comparison of **(A)** hsa-miR-34a-5p, **(B)** hsa-miR-106b-5p, **(C)** hsa-miR-26a-5p and **(D)** hsa-miR-20a-5p expression in untreated and bleomycin-treated WT, AT648, AT5849 and AT9607 fibroblast lines. For each cell line, expression levels were normalized to hsa-miR-16-5p and presented relative to the corresponding untreated condition. Statistical analysis was performed on ΔCt values using paired Student’s t-tests between untreated and bleomycin-treated matched biological replicates. Bars represent mean ± SD from three independent biological replicates. *p < 0.05, **p < 0.01, ***p < 0.001; ns, not significant

The between-line comparisons showed that A-T fibroblast retained altered miRNA expression after bleomycin exposure but did not determine whether each line mounted a within-line response to genotoxic stress. We therefore compared untreated and bleomycin-treated samples within each cell line using paired analyses on ΔCt values (Fig. 3). In WT fibroblast, bleomycin induced significant miR-34a-5p expression (*p* = 0.01) and reduced miR-106b-5p expression significantly (*p* < 0.001). miR-26a-5p showed an upward change that did not reach statistical significance (*p* = 0.05), whereas miR-20a-5p was unchanged (*p* = 0.50). This pattern suggests a selective stress-associated response rather than broad induction of the full candidate panel. AT648 showed the most pronounced and coordinated response to bleomycin. All four candidate miRNAs were significantly induced after treatment: miR-34a-5p (*p* = 0.005), miR-106b-5p (*p* = 0.001), miR-26a-5p (*p* = 0.02) and miR-20a-5p (*p* = 0.006). Thus, AT648 was not inert to bleomycin exposure, but instead displayed broad stress-associated miRNA induction. Importantly, this within-line induction did not restore miR-34a-5p or miR-26a-5p to WT-like levels in the between-line analysis. AT5849 showed a distinct response profile. miR-34a-5p showed a non-significant trend after bleomycin treatment (*p* = 0.06), while miR-106b-5p was significantly reduced (*p* = 0.03). miR-26a-5p and miR-20a-5p were unchanged (*p* = 0.86 and *p* = 0.59, respectively). In AT9607, bleomycin significantly induced miR-34a-5p (*p* < 0.001), whereas miR-106b-5p, miR-26a-5p and miR-20a-5p did not change significantly (*p* = 0.26, *p* = 0.78 and *p* = 0.84, respectively). Overall, bleomycin responsiveness was highly cell-line-specific. WT, AT648 and AT9607 showed miR-34a-5p inducibility, but only AT648 displayed coordinated induction of all four tested miRNAs. Thus, the present data do not define a uniform A-T-specific bleomycin-response signature; rather, treatment responsiveness was heterogeneous across the individual fibroblast lines. Within-cell-line statistical comparisons are provided in Supplementary Table S2.

### Target-based enrichment characterizes pathways represented by the predefined candidate miRNA panel

**Fig. 4:**
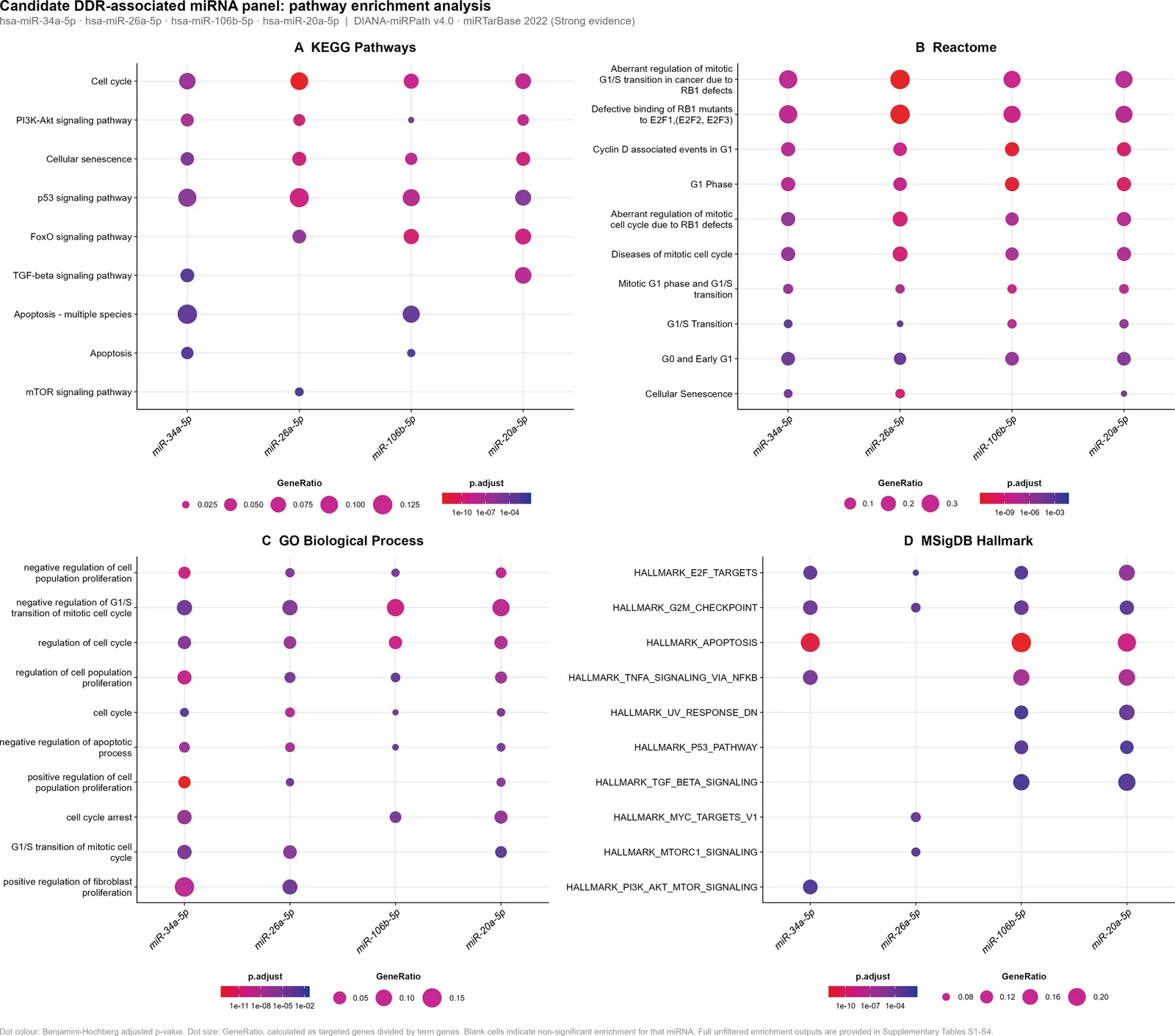
Target-based enrichment analysis of the candidate DDR-associated miRNA panel. DIANA-miRPath v4.0 was used to perform miRNA-centric functional enrichment analysis of hsa-miR-34a-5p, hsa-miR-26a-5p, hsa-miR-106b-5p and hsa-miR-20a-5p using experimentally validated miRNA-target interactions from miRTarBase 2022. hsa-miR-16-5p was excluded because it was used as the endogenous normalizer. Enrichment results are shown for **(A)** KEGG pathways, **(B)** Reactome pathways, **(C)** Gene Ontology Biological Process terms and **(D)** MSigDB Hallmark gene sets. Dot colour represents the Benjamini-Hochberg FDR-adjusted p-value, and dot size represents the GeneRatio, calculated as the number of targeted genes divided by the total number of genes in the corresponding term. Blank cells indicate non-significant enrichment for the corresponding miRNA or gene-set category. Significant terms relevant to DNA damage response, cell-cycle regulation, checkpoint control, apoptosis, senescence and survival signalling were prioritized for visualization. Because the miRNAs were selected a priori for their reported relationships with DDR and checkpoint biology, the enrichment results are interpreted as functional annotation of the predefined candidate panel rather than independent validation of the RT-qPCR findings. Full unfiltered enrichment outputs are provided in Supplementary Tables S3 to S6

To explore the biological processes potentially associated with the candidate miRNA panel, target-based enrichment analysis was performed using DIANA-miRPath v4.0 with miRTarBase 2022 target annotations as the primary experimentally validated interaction resource. All four candidate miRNAs were retained in this analysis regardless of the magnitude or statistical significance of their individual RT-qPCR differences because the objective was to characterize the target space of the prespecified panel rather than to perform enrichment of an unbiased set of differentially expressed miRNAs. Across KEGG, Reactome, GO Biological Process and MSigDB Hallmark analyses, the displayed biologically prioritized significant terms converged on pathways related to cell-cycle regulation, G1/S transition, RB/E2F signaling, p53-associated stress response, apoptosis, senescence and survival signaling (Fig. 4). KEGG enrichment highlighted Cell cycle, PI3K-Akt signaling pathway, Cellular senescence, p53 signaling pathway, FoxO signaling pathway, TGF-beta signaling pathway, Apoptosis and mTOR signaling pathway. Reactome enrichment strongly emphasized G1-phase and RB/E2F-associated biology, including Cyclin D associated events in G1, G1 Phase, Mitotic G1 phase and G1/S transition, G1/S Transition and RB1/E2F-related G1/S transition terms. GO Biological Process enrichment included regulation of cell cycle, negative regulation of G1/S transition of the mitotic cell cycle, cell-cycle arrest, negative regulation of apoptotic process and proliferation-associated terms. DNA-damage checkpoint-related terms were also present in the full GO Biological Process output. MSigDB Hallmark enrichment further supported this pattern through enrichment of E2F Targets, G2M Checkpoint, Apoptosis, p53 Pathway, TGF-beta Signaling, mTORC1 Signaling and PI3K-AKT-mTOR Signaling gene sets. Full DIANA-miRPath miRTarBase 2022 enrichment outputs are provided in Supplementary Tables S3 to S6. DIANA-TarBase v8.0 was additionally used as a sensitivity interaction resource and produced a broadly overlapping pathway-level pattern, particularly for cell-cycle, checkpoint, senescence, p53-associated and stress-response pathways. Full sensitivity outputs are provided in Supplementary Tables S10 to S13. Because the four miRNAs were selected partly on the basis of reported relationships with p53, checkpoint, and cell-cycle biology, recovery of related pathway annotations is expected and should not be interpreted as independent confirmation of the RT-qPCR findings or as evidence that these pathways are functionally altered in the analysed fibroblast samples. The enrichment analyses are used here only to organize candidate biological contexts for subsequent experimental testing.

Complementary MIENTURNET functional enrichment of the shared miRNA-target network recovered a broadly overlapping pathway structure, including cell-cycle regulation, G1/S control, cellular senescence, p53 signaling, apoptosis, PI3K-Akt signaling and DNA-damage-associated terms. These complementary outputs support the interpretation that the predefined candidate miRNA panel is primarily linked to G1/S control, RB/E2F regulation, p53-associated stress signaling, apoptosis, senescence and DNA-damage-associated processes. Complementary MIENTURNET KEGG and Reactome enrichment outputs are provided in Supplementary Tables S8 and S9.

### Network analysis identifies shared candidate target nodes involved in checkpoint and survival regulation

**Fig. 5:**
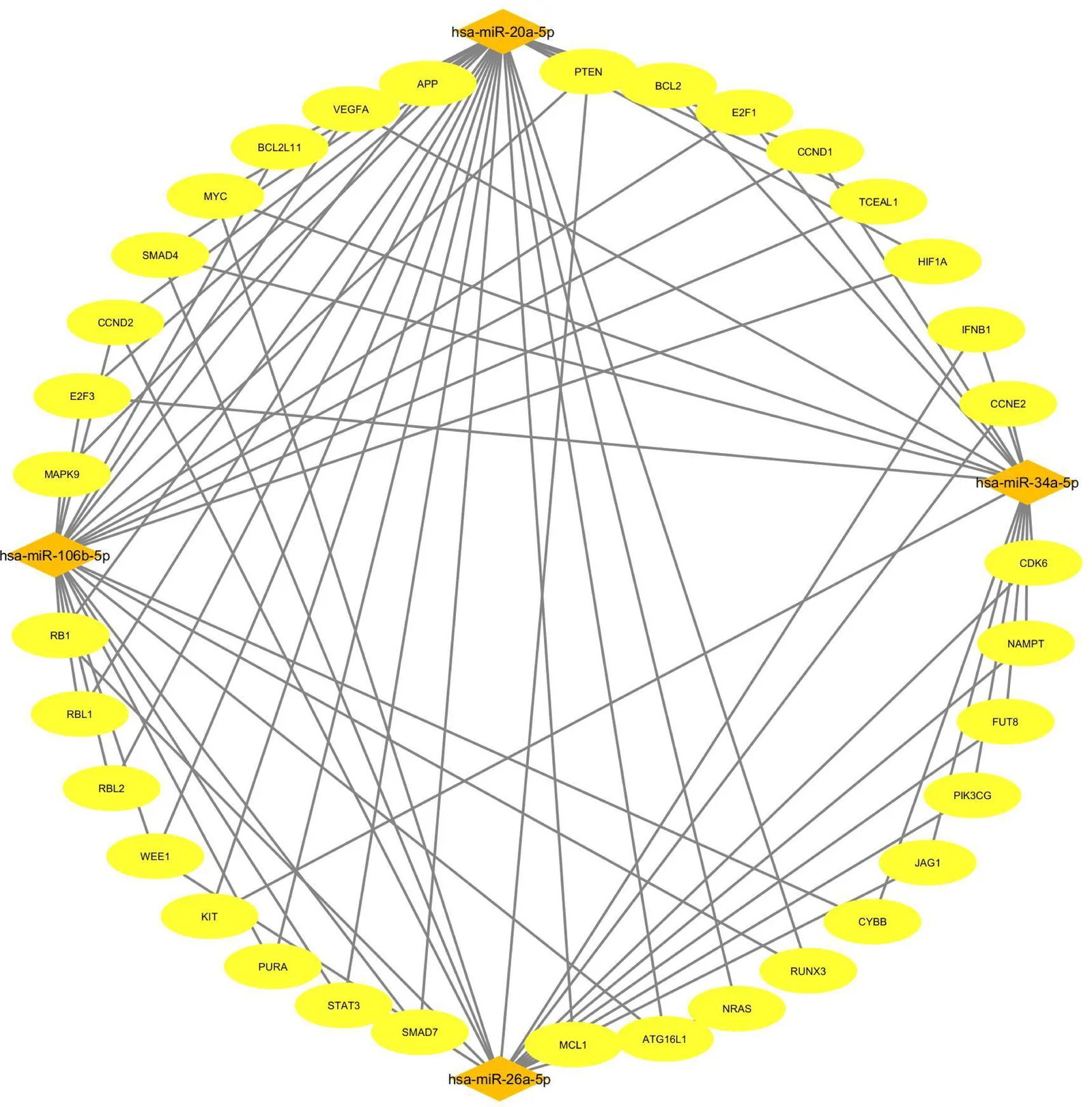
miRNA-target interaction network of the candidate DDR-associated miRNA panel. MIENTURNET was used to retrieve experimentally supported miRNA-target interactions from miRTarBase for hsa-miR-34a-5p, hsa-miR-26a-5p, hsa-miR-106b-5p and hsa-miR-20a-5p. hsa-miR-16-5p was excluded because it was used as the endogenous normalizer. The exported interaction table was visualized in Cytoscape after retaining target genes shared by at least two input miRNAs. miRNA nodes and target-gene nodes are displayed as distinct node classes. The network highlights shared candidate regulatory targets associated with G1/S progression, RB/E2F signalling, checkpoint regulation, apoptosis and survival-related pathways. This network is hypothesis-generating and does not demonstrate direct target regulation in the analysed fibroblast samples

MIENTURNET was used to identify experimentally supported miRNA-target interactions shared by the four candidate miRNAs. In the miRTarBase-based network output, no target gene was shared by all four input miRNAs. Ten genes were connected to three miRNAs: *CCND1*,* CCND2*,* E2F1*,* E2F3*,* MYC*,* PTEN*,* RB1*,* SMAD4*,* VEGFA* and *WEE1*. A further 24 genes were connected to exactly two candidate miRNAs. hsa-miR-106b-5p and hsa-miR-20a-5p shared APP, ATG16L1, BCL2L11, HIF1A, MAPK9, PURA, RBL1, RBL2, RUNX3, SMAD7, STAT3, and TCEAL1. hsa-miR-34a-5p and hsa-miR-26a-5p shared CCNE2, CDK6, FUT8, IFNB1, JAG1, NAMPT, and PIK3CG. hsa-miR-20a-5p and hsa-miR-34a-5p shared BCL2 and KIT; hsa-miR-20a-5p and hsa-miR-26a-5p shared MCL1 and NRAS; and hsa-miR-106b-5p and hsa-miR-34a-5p shared CYBB. Shared targets mapped to G1/S progression, RB/E2F control, checkpoint regulation, apoptosis and survival signalling. *WEE1* was notable because it links the network to checkpoint kinase regulation. The presence of *E2F1*,* E2F3*,* RB1*,* CCND1*,* CCND2* and *CDK6* supported convergence on the RB/E2F and G1/S axis, whereas *BCL2* and *BCL2L11* linked the panel to apoptosis-associated regulation. *PTEN*,* MYC*,* VEGFA*,* SMAD4* and *STAT3* further connected the network to survival, proliferative and stress-response signalling. These network findings provide a candidate mechanistic framework for interpreting the qPCR results, but they remain predictive (Fig. 5). Direct measurement of target mRNA and protein expression will be required to determine whether these candidate miRNA-target relationships are active in the analysed A-T fibroblast panel. The network therefore provides a hypothesis-generating framework for prioritizing candidate miRNA-target relationships. Although the included interactions are supported by the underlying interaction database, their activity was not experimentally established in the fibroblast samples analysed here. Direct measurement of target mRNA and protein abundance would be required to determine whether these relationships are functionally relevant in this experimental system. The full MIENTURNET miRNA-target interaction network is provided in Supplementary Table S7.

## Discussion

The present study identifies a selective DDR-associated miRNA phenotype in A-T fibroblast panel, characterized by reproducible basal depletion of miR-34a-5p and miR-26a-5p, persistent failure of these two miRNAs to reach WT-like levels after bleomycin exposure, and cell-line-specific remodeling of miR-106b-5p and miR-20a-5p under genotoxic stress. The A-T lines were not globally miRNA non-responsive after genotoxic stress; rather, they showed persistent failure to normalize miR-34a-5p and miR-26a-5p to WT-like levels, while miR-106b-5p and miR-20a-5p behaved in a more condition-dependent manner. AT648 illustrated this most clearly, with coordinated induction of all four tested miRNAs after bleomycin despite persistent reduction of miR-34a-5p and miR-26a-5p relative to bleomycin-treated WT. Thus, the main biological message is the coexistence of shared basal miRNA depletion and abnormal post-damage remodeling. These findings therefore describe candidate expression patterns in the present fibroblast panel rather than a uniform or disease-wide A-T miRNA signature. These findings should be interpreted within a still limited A-T ncRNA literature. A recent scoping review showed that only a small number of primary studies have examined ncRNA dysregulation in A-T, with available evidence spanning baseline miRNA dysregulation, radiation-responsive miRNA behavior, and ATM-dependent lncRNA induction. That review also emphasized that A-T ncRNA behavior differs by cell type, stimulus, dose, and time point, rather than forming a single uniform signature [31].

The present study is complementary to the recent A-T miRNA profiling work of Cirillo et al. rather than a replication of it. Cirillo et al. provided the strongest disease-level baseline profiling study, identifying 42 differentially expressed miRNAs in A-T PBMCs and 26 in fibroblasts, followed by validation of miR-195-5p, miR-30a-5p and miR-342-3p in additional samples [22]. Bryant et al. instead examined ATM-null lymphoblastoid cell lines after low-dose irradiation and reported bidirectional miRNA changes, with both upregulated and downregulated species [32]. Kabacik et al. showed that miR-34a-5p and miR-182-5p increased after irradiation in stimulated T lymphocytes, but this response was largely not discriminatory between A-T and control samples [33]. Smirnov et al. further separated irradiation-responsive miRNAs into recessive and dominant expression patterns in EBV-transformed lymphoblastoid cell lines [34]. Together, these studies show that A-T miRNA biology is model-dependent, stimulus-dependent and time-dependent, rather than reducible to a single disease-wide miRNA signature. The present study adds a different layer to this field by testing a predefined ATM, p53, G1/S and DDR-linked candidate panel in A-T fibroblast models under controlled bleomycin exposure.

Among the four tested miRNAs, miR-34a-5p provides the clearest mechanistic link to the ATM-p53 checkpoint axis. ATM is an upstream regulator of p53 activation after DNA damage, and miR-34a is a direct p53-responsive miRNA involved in apoptosis, cell-cycle arrest and tumor-suppressive stress responses [11–14, 35–37]. Therefore, the consistent basal depletion of miR-34a-5p across all three A-T fibroblast lines relative to the single control is biologically coherent with impaired ATM-p53 signaling. However, the important point is not merely that miR-34a-5p belongs to the p53 network. The stronger observation is that miR-34a-5p depletion was reproducible across A-T fibroblast backgrounds and persisted after bleomycin exposure, indicating that DNA damage did not restore the single control WT-like miR-34a-5p abundance. This interpretation is strengthened by evidence that miR-34a can be regulated beyond transcription. Salzman et al. showed that DNA damage can regulate mature miR-34 activity through ATM-dependent 5′-end phosphorylation, independently of de novo p53-mediated transcription [15]. In parallel, ATM has also been linked to broader miRNA processing through phosphorylation of KSRP, suggesting that ATM loss may affect miRNA regulation not only through p53-dependent transcription but also through post-transcriptional miRNA biogenesis mechanisms [9]. Thus, ATM deficiency could affect miR-34a biology at both transcriptional and mature-miRNA activity levels. However, the present study measured mature miR-34a-5p abundance only, not miR-34a phosphorylation, AGO loading or target repression. The data therefore support altered miR-34a-5p abundance, while impaired miR-34a activity remains a mechanistic hypothesis for future testing.

The bleomycin response of miR-34a-5p adds an important nuance. The WT, AT648 and AT9607 all showed within-line miR-34a-5p inducibility after bleomycin, but the A-T lines remained below bleomycin-treated WT. Therefore, the data support impaired normalization rather than complete absence of inducibility. This distinction is important when comparing the present results with earlier radiation-response studies. Kabacik et al. reported that miR-34a-5p increased after irradiation in stimulated human T lymphocytes, but the response was not strongly discriminatory between A-T and control samples [33]. The present fibroblast data suggest a different pattern as miR-34a-5p can remain inducible in some A-T lines yet still fail to reach the single control WT-like abundance after DNA damage. This difference may reflect cell type, damage modality, sampling time, transformation status or recovery duration. Since ATR, DNA-PK and other compensatory pathways were not measured here, residual miR-34a-5p inducibility should be interpreted cautiously as evidence of partial stress responsiveness, not as restoration of normal ATM-dependent regulation.

miR-26a-5p extends the phenotype beyond the canonical miR-34a axis and should be treated as the second major result of the study. It was reduced in all three A-T lines at baseline and remained lower than the single control WT after bleomycin exposure, making it one of the most reproducible alterations in the panel. However, significant within-line induction of miR-26a-5p was not observed consistently across the panel. Its principal finding in the present study is therefore the persistent between-line difference relative to the single control rather than a uniform bleomycin-responsive phenotype. Lezina et al. linked miR-26a to p53-associated genotoxic stress and to checkpoint kinases including WEE1 and CHK1 [16] while other studies connect miR-26a to PTEN-AKT and DNA repair-associated phenotypes in cancer models [17, 18]. These studies justify interpreting miR-26a-5p as a DDR-associated candidate, but they do not prove that WEE1, CHK1, PTEN or other targets are regulated by miR-26a-5p in the present study. The AT648 response again separates inducibility from normalization: miR-26a-5p increased after bleomycin within AT648, yet remained below bleomycin-treated WT. Thus, miR-26a-5p depletion nominates WEE1, CHK1 and related checkpoint axes for validation.

Furthermore, AT648 was the most distinctive A-T line and should be interpreted as biologically informative, while still recognizing that its response may be influenced by cell-line-specific genotype, immortalization history, basal stress state or residual compensatory signaling. It was the only line in which bleomycin induced all four measured miRNAs. This pattern differed from WT, where bleomycin induced miR-34a-5p but reduced miR-106b-5p, and from AT9607, where only miR-34a-5p was clearly induced. The AT648 response is also functionally complex because the induced miRNAs do not point in a single direction: miR-34a-5p and miR-26a-5p are linked to p53, checkpoint and stress-response biology, whereas miR-106b-5p and miR-20a-5p are linked to p21, E2F and G1/S regulatory modules [9, 11–21]. Coordinated induction of all four miRNAs after bleomycin therefore should not be interpreted as a clean pro-arrest or pro-repair response. It is better interpreted as abnormal stress-induced remodeling at the checkpoint/proliferation interface.

The AT648-specific induction of miR-106b-5p and miR-20a-5p nominates a p21/RB/E2F checkpoint hypothesis. miR-106b family members can regulate CDKN1A/p21 and promote cell-cycle progression [19]. Since p21 is a major p53 effector controlling G1/S arrest after DNA damage [38], induction of miR-106b-5p in an ATM-deficient background could plausibly weaken checkpoint restraint. miR-20a-5p adds a parallel E2F-linked layer. O’Donnell et al. showed that c-Myc-regulated miRNAs, including miR-20a, modulate E2F1 [20, 39]. This is particularly relevant because E2F1 is not merely a proliferation gene; E2F1 signaling intersects functionally with ATM-dependent p53 phosphorylation and apoptosis. In primary human fibroblasts lacking functional ATM, E2F1-induced p53 phosphorylation and apoptosis were impaired, supporting a direct functional connection between the RB/E2F axis and ATM-dependent cell-fate control [40].

**Fig. 6:**
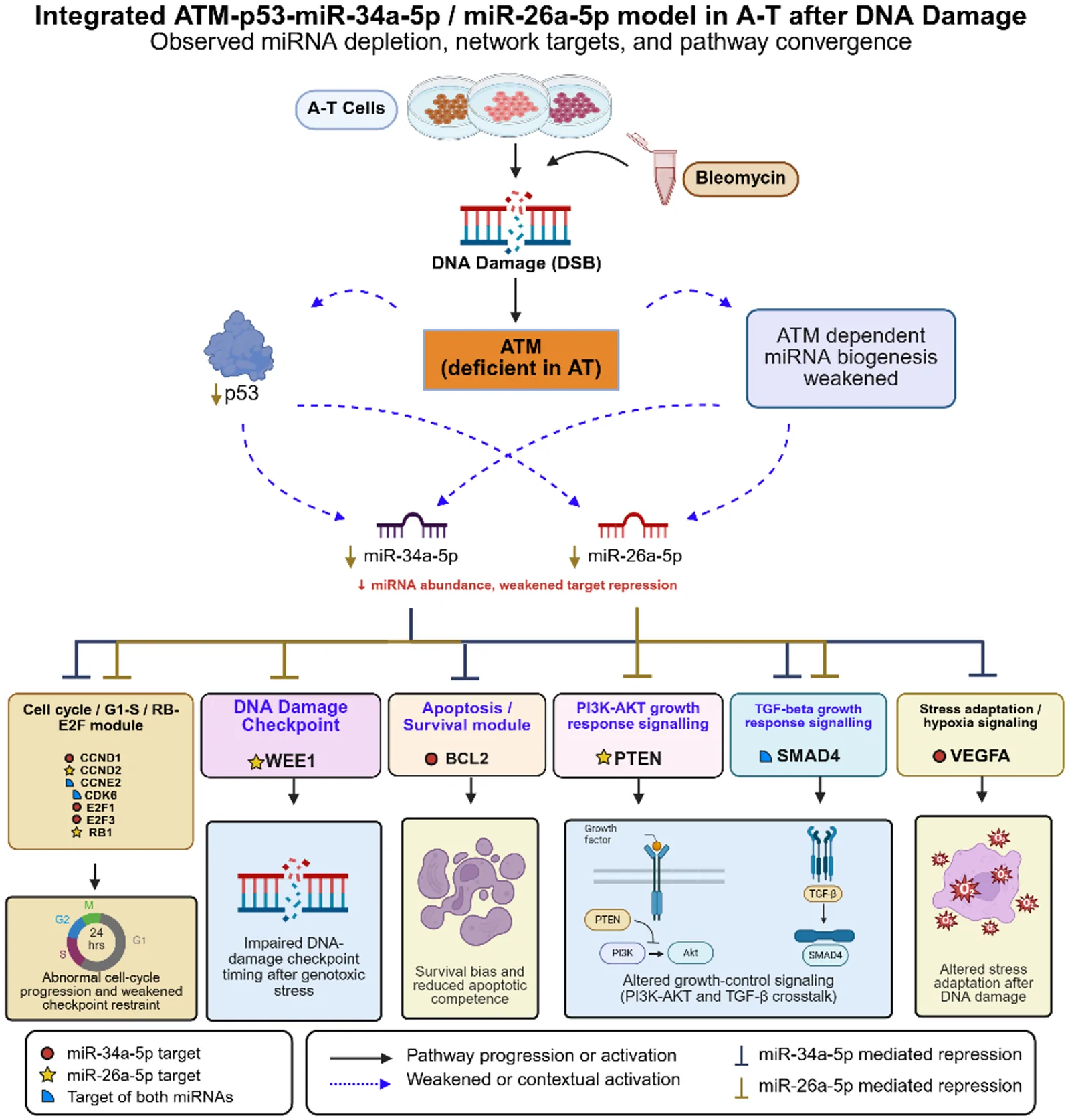
Proposed model linking persistent miR-34a-5p/miR-26a-5p depletion to altered regulatory pressure on ATM-relevant target modules after bleomycin exposure. Bleomycin-induced DNA double-strand breaks in A-T fibroblast lines occur in the context of defective ATM signaling, which may compromise downstream p53-associated stress responses and ATM-linked regulation of DDR-associated miRNAs. In this model, persistent depletion of hsa-miR-34a-5p and hsa-miR-26a-5p may reduce predicted miRNA-mediated repression pressure on experimentally supported candidate target modules. hsa-miR-34a-5p associated targets, including *CCND1*,* CDK6*,* E2F1*,* E2F3*,* BCL2* and *VEGFA*, converge mainly on G1/S transition, RB/E2F signaling, apoptosis/survival balance and stress-adaptation pathways. hsa-miR-26a-5p associated targets, including *WEE1*,* CCND2*,* CCNE2*,* RB1*,* PTEN* and *SMAD4*, converge on DNA-damage checkpoint timing, G1/S regulation, PI3K-AKT signaling and TGF-beta growth-response pathways. Together, depletion of hsa-miR-34a-5p and hsa-miR-26a-5p may reduce predicted repression pressure on candidate target modules that are involved in cell-cycle progression, checkpoint timing, survival and stress-response signaling. Solid black arrows indicate pathway progression or activation, dotted blue arrows indicate weakened or context-dependent activation, and T-bars indicate literature supported miRNA-mediated repression. T-bars indicate repression of the listed target genes within each module, not direct inhibition of the entire pathway. All miRNA-target gene relationships shown are based on experimentally supported interactions from miRTarBase 2022 and MIENTURNET network analysis. Target-gene expression and protein-level regulation were not experimentally validated in the present study

The enrichment and network analyses should be interpreted as hypothesis-generating annotations of the predefined candidate panel rather than as independent support for the RT-qPCR findings. The four miRNAs were selected a priori partly because of their reported involvement in p53, cell-cycle, checkpoint, and DNA damage-associated biology; therefore, enrichment of these same biological processes among their documented targets is expected and does not provide independent confirmation of an ATM-associated pathway phenotype. Across DIANA-miRPath and MIENTURNET outputs, the documented targets of the four-miRNA candidate panel were annotated to biological processes including cell-cycle regulation, G1/S transition, RB/E2F signaling, p53 signaling, apoptosis, senescence, PI3K-Akt/mTOR signaling, TGF-beta signaling and DNA-damage-associated processes. This pattern is consistent with the selected biology of the panel and with the pathway-level enrichment reported by Cirillo et al. for dysregulated A-T miRNAs [22]. Rather than interpreting individual target genes in isolation, the network can be organized into five interrelated candidate modules. First, the RB/E2F and G1/S module, including *CCND1*,* CCND2*,* CCNE2*,* CDK6*,* RB1*,* RBL1*,* RBL2*,* E2F1*,* E2F3* and *MYC*, represents a prominent candidate connection between the measured miRNAs and cell-cycle control. This module is especially relevant to A-T because ATM-p53 signaling normally contributes to checkpoint restraint after DNA damage, while E2F1 can also engage ATM-dependent p53 and CHK2 phosphorylation and apoptosis [40]. Second, a checkpoint-kinase module, including WEE1 and the literature-supported nodes CHK1, CHK2 and CDKN1A/p21, identifies checkpoint timing and checkpoint regulation as candidate areas for subsequent functional investigation. Third, an apoptosis/survival module, including *BCL2*, *BCL2L11* and *MCL1*, connects the miRNA panel to cell-fate regulation. Fourth, the PTEN, PI3K-Akt and mTOR module links the network to survival, growth and stress-adaptation signaling, and is independently relevant to ATM biology because ATM-mediated PTEN phosphorylation has been reported to promote PTEN nuclear translocation and autophagy after DNA damage [41]. Fifth, the TGF-beta/SMAD and hypoxia/stress module, including SMAD4, SMAD7, STAT3, HIF1A and VEGFA, broadens the network toward growth-response and stress-adaptive signaling; this is biologically plausible because DNA damage can activate TGF-beta signaling through an ATM-c-Cbl-TbetaRII axis and Smad proteins have been linked to ATM-associated DNA damage signaling [42, 43]. Importantly, these modules remain candidate mechanisms. The present study measured mature miRNA abundance, not target mRNA, protein expression or pathway activity. Therefore, the enrichment and network analyses should be viewed as a testable map of ATM-relevant target modules that may be affected by miR-34a-5p/miR-26a-5p depletion and by cell-line-specific miR-106b-5p/miR-20a-5p remodeling, rather than as causal evidence of target-gene dysregulation in the analysed fibroblast samples. This interpretation is summarized in the proposed model shown in Fig. 6. The model is intentionally hypothesis-generating: it integrates measured miRNA abundance with literature-supported target modules, but target-gene expression, protein abundance and pathway activity remain to be experimentally validated.

Several limitations define the interpretation boundary of this study. First, only one ATM-proficient control line was included, and each of the three A-T cell lines represents an individual donor. Consequently, donor-specific or cell-line-specific effects cannot be separated definitively from differences associated with A-T. The biological replicates provide replication of the experimental measurements within each cell line. The between-line comparisons should therefore be interpreted as findings within this fibroblast panel rather than as population-level estimates of A-T-specific miRNA dysregulation. Second, the sample size was limited to three biological replicates per cell line and condition, which restricts power for variable miRNAs such as miR-20a-5p. In addition, no multiplicity correction was applied across the complete set of miRNAs, cell lines, and experimental conditions; the reported inferential analyses should therefore be interpreted as exploratory. Third, miR-16-5p was used as a single endogenous normalizer. Although miR-16-5p is commonly used in miRNA RT-qPCR normalization studies [44, 45], a single reference miRNA is less robust than a geometric mean of multiple validated references, and miR-16 itself has been linked to p53-associated genotoxic stress responses [16]. Therefore, treatment- or cell-line-associated variation in the endogenous normalizer cannot be excluded and represents a limitation of the relative-expression analysis. Fourth, the experiment used one bleomycin dose and one recovery time point. miRNA responses can be acute, transient or delayed, so a 24 h recovery design captures only one phase of the response. Fifth, mature miRNA abundance was measured, but pri-miRNA, pre-miRNA, AGO loading, miRNA phosphorylation and target repression were not measured. Sixth, database derived candidate targets were not validated at mRNA or protein level. Therefore, *WEE1*,* CHK1*,* RB1*,* E2F1*,* E2F3*,* CCND1*,* CCND2*,* CDK6*,* BCL2*,* BCL2L11*,* PTEN*,* MYC* and *VEGFA* should be described as candidate nodes, not confirmed effectors. Finally, differences in cell-line background, including immortalization or transformation status, may affect p53 and RB/E2F biology and therefore may contribute to line-specific responses.

A priority for future work is target-level and phenotype-level validation of the checkpoint model proposed here. *p53* activation, *p21/CDKN1A*,* WEE1*,* CHK1*,* RB1*,* E2F1*,* E2F3*,* cyclin D1*, cyclin *D2*, *CDK6*,* BCL2*,* BCL2L11* and *PTEN* should be assessed at mRNA and protein levels under the same untreated and bleomycin-treated conditions. Based on the expanded network interpretation, *SMAD4*,* SMAD7*,* STAT3*,* HIF1A* and *VEGFA* could be added as second-tier validation targets if the study is extended toward TGF-beta, inflammatory or stress-adaptation modules. AT648 should be prioritized because it shows the clearest abnormal miRNA remodelling phenotype. Cell-cycle profiling after bleomycin would directly test whether AT648-specific induction of miR-106b-5p and miR-20a-5p is associated with altered G1/S checkpoint behaviour. miRNA mimic and inhibitor experiments would be needed to establish causality. The strongest causal experiment would be to restore miR-34a-5p or miR-26a-5p in selected A-T fibroblast lines, then test whether candidate targets and checkpoint phenotypes shift in the expected direction. Matched transcriptomic data would further allow identification of targets inversely associated with measured miRNA changes, but such integration should be treated as supportive unless performed in the same biological samples. Overall, these findings support a checkpoint-centered model in which A-T fibroblast lines show stable depletion of selected ATM, p53 and DDR-associated miRNAs, together with cell-line-specific stress-induced remodeling of G1/S and RB/E2F-associated miRNA networks. The model remains hypothesis-generating, but it provides a clear framework for future experiments that distinguish miRNA inducibility, target derepression and true restoration of WT-like checkpoint regulation.

## Conclusion

This study identifies a selective DDR-associated miRNA phenotype in a fibroblast panel comprising three A-T lines and one WT control line. The most consistent abnormality was basal depletion of miR-34a-5p and miR-26a-5p, with both miRNAs remaining below WT levels after bleomycin exposure. However, bleomycin responsiveness was not uniformly absent: AT648 and AT9607 retained miR-34a-5p inducibility, and AT648 showed coordinated induction of all four tested miRNAs. Together with pathway and network analyses, these findings support a checkpoint-centered model in which A-T fibroblast lines show shared basal disruption of selected ATM, p53 and DDR-associated miRNAs, combined with cell-line-specific remodeling of G1/S and RB/E2F-associated miRNA networks. This model remains hypothesis-generating and requires validation at the target mRNA, protein and functional checkpoint levels.

## Supplementary Information

Below is the link to the electronic supplementary material.

Supplementary Material 1

Supplementary Material 2

## Data Availability

Statistical comparisons, enrichment outputs and MIENTURNET outputs are provided in Supplementary File S1 and S2, Tables S1 to S13. Supplementary Files provide descriptions of the supplementary tables.

## Declarations

### Generative AI and AI-assisted technologies in the writing process

During the preparation of this work, the authors used ChatGPT to support language editing, clarity improvement and manuscript organization. The authors reviewed and edited all content and take full responsibility for the final manuscript.
