# Supplementary material for: DNA damage response associated miRNA dysregulation in Ataxia-telangiectasia
Source: Mol Biol Rep. 2026 Sep 30;53(1):1660. doi: 10.1007/s11033-026-12860-1 (PMC13627277; doi:10.1007/s11033-026-12860-1)
Supplement: Supplementary file 1 [file 11033_2026_12860_MOESM1_ESM.docx]

**Supplementary File S1: Information of all Supplementary Tables**

- Table S1. Complete statistical analysis summary
  - S1A. Baseline, ANOVA + Dunnett's vs untreated WT
  - S1B. Bleomycin-treated, ANOVA + Dunnett's vs bleomycin-treated WT
- Table S2. Complete statistical analysis summary of within-cell-line bleomycin response, paired t-test on ΔCt values

**Table S1A. Baseline, One Way ANOVA + Dunnett's vs untreated WT**

| **miRNA** | **Comparison** | **Adj. p-value** | **Sig.** |
| --- | --- | --- | --- |
| miR-34a-5p | WT− vs AT648− | p < 0.001 | *** |
| miR-34a-5p | WT− vs AT5849− | p < 0.001 | *** |
| miR-34a-5p | WT− vs AT9607− | p < 0.001 | *** |
| miR-26a-5p | WT− vs AT648− | p = 0.001 | ** |
| miR-26a-5p | WT− vs AT5849− | p = 0.003 | ** |
| miR-26a-5p | WT− vs AT9607− | p = 0.001 | ** |
| miR-106b-5p | WT− vs AT648− | p = 0.03 | * |
| miR-106b-5p | WT− vs AT5849− | p = 0.18 | ns |
| miR-106b-5p | WT− vs AT9607− | p = 0.01 | * |
| miR-20a-5p | WT− vs AT648− | p = 0.21 | ns |
| miR-20a-5p | WT− vs AT5849− | p = 0.32 | ns |
| miR-20a-5p | WT− vs AT9607− | p = 0.37 | ns |

**Table S1B. Bleomycin-treated, ANOVA + Dunnett's vs bleomycin-treated WT**

| **miRNA** | **Comparison** | **Adj. p-value** | **Sig.** |
| --- | --- | --- | --- |
| miR-34a-5p | WT+ vs AT648+ | p < 0.001 | *** |
| miR-34a-5p | WT+ vs AT5849+ | p < 0.001 | *** |
| miR-34a-5p | WT+ vs AT9607+ | p < 0.001 | *** |
| miR-26a-5p | WT+ vs AT648+ | p < 0.001 | *** |
| miR-26a-5p | WT+ vs AT5849+ | p < 0.001 | *** |
| miR-26a-5p | WT+ vs AT9607+ | p < 0.001 | *** |
| miR-106b-5p | WT+ vs AT648+ | p < 0.001 | *** |
| miR-106b-5p | WT+ vs AT5849+ | p = 0.91 | ns |
| miR-106b-5p | WT+ vs AT9607+ | p = 0.27 | ns |
| miR-20a-5p | WT+ vs AT648+ | p = 0.03 | * |
| miR-20a-5p | WT+ vs AT5849+ | p = 0.65 | ns |
| miR-20a-5p | WT+ vs AT9607+ | p = 0.04 | * |

**Table S2. Within-cell-line bleomycin response, paired t-test on ΔCt values**

| **Cell line** | **miRNA** | **p-value** | **Sig.** |
| --- | --- | --- | --- |
| WT | miR-34a-5p | p = 0.01 | * |
| WT | miR-106b-5p | p < 0.001 | *** |
| WT | miR-26a-5p | p = 0.05 | ns |
| WT | miR-20a-5p | p = 0.5 | ns |
| AT648 | miR-34a-5p | p = 0.005 | ** |
| AT648 | miR-106b-5p | p = 0.001 | ** |
| AT648 | miR-26a-5p | p = 0.02 | * |
| AT648 | miR-20a-5p | p = 0.006 | ** |
| AT5849 | miR-34a-5p | p = 0.06 | ns |
| AT5849 | miR-106b-5p | p = 0.03 | * |
| AT5849 | miR-26a-5p | p = 0.86 | ns |
| AT5849 | miR-20a-5p | p = 0.59 | ns |
| AT9607 | miR-34a-5p | p < 0.001 | *** |
| AT9607 | miR-106b-5p | p = 0.26 | ns |
| AT9607 | miR-26a-5p | p = 0.78 | ns |
| AT9607 | miR-20a-5p | p = 0.84 | ns |
